# The Efficacy of Manual Therapy and Pressure Biofeedback-Guided Deep Cervical Flexor Muscle Strength Training on Pain and Functional Limitations in Individuals with Cervicogenic Headaches: A Randomized Comparative Study

**DOI:** 10.1155/2023/1799005

**Published:** 2023-08-14

**Authors:** Shahnaz Hasan, Nasrin Bharti, Ahmad H. Alghadir, Amir Iqbal, Naiyer Shahzad, Abeer R. Ibrahim

**Affiliations:** ^1^Department of Physical Therapy and Health Rehabilitation, College of Applied Medical Sciences, Majmaah University, Al-Majmaah 11952, Saudi Arabia; ^2^Department of Physiotherapy, Buddha Paramedical College, GIDA, Gorakhpur, UP 273209, India; ^3^Department of Rehabilitation Sciences, College of Applied Medical Sciences, King Saud University, Riyadh 11433, Saudi Arabia; ^4^Department of Pharmacology and Toxicology, College of Medicine, Umm Al-Qura University, Makkah 21955, Saudi Arabia; ^5^Department of Physiotherapy, College of Applied Medical Sciences, Umm Al-Qura University, Makkah 21955, Saudi Arabia; ^6^Department of Basic Science, Faculty of Physical Therapy, Cairo University, Giza 12613, Egypt

## Abstract

**Objective:**

This study aimed to compare the efficacy of manual therapy and pressure biofeedback-guided DCFM strength training on pain intensity and functional limitations in individuals with CGH. *Trial Design*. A double-blinded, two-arm parallel group randomized comparative design.

**Methods:**

After applying the eligibility criteria, sixty out of eighty-nine CGH patients were recruited from King Saud University Medical Center in Riyadh and randomly allocated to intervention groups using simple random sampling. Group 1 underwent pressure biofeedback-guided DCFM strength training and conventional treatment, while Group 2 received manual therapy and conventional treatment for three consecutive weeks. The main outcome measures were scores on the visual analog scale (VAS) and the headache disability index (HDI). One assessor and two physical therapists were blinded to group allocation.

**Results:**

Sixty out of eighty participants aged 29–40 years were randomized into intervention groups (*n* = 30/group; age (mean ± standard deviation): group 1 = 35.0 ± 2.82; group 2 = 34.87 ± 2.60), and their data were analyzed. A significant improvement (95% CI, *p* < 0.05) was observed within each group when comparing the VAS and HDI scores between baseline and postintervention. In contrast, between-group comparisons for the outcome score of VAS and HDI revealed nonsignificant differences in the first, second, and third weeks after intervention, except for the VAS score, which showed a significant difference in weeks 2 and 3 after intervention. Cohen's *d*-value indicated that the intervention effect size for reducing pain was larger in group 1 than in group 2 at weeks 2 and 3.

**Conclusion:**

Compared with manual therapy, pressure biofeedback-guided DCFM strength training showed a greater reduction in pain intensity (assessed using the VAS) at weeks two and three. However, both treatments were equally effective in lowering headache-related functional limitations in patients with CGH. This trial is registered with ClinicalTrial.gov PRS (Identifier ID: NCT05692232).

## 1. Introduction

Cervicogenic headache (CGH) is unilateral pain that starts in the neck and is referred from bony structures or soft tissues of the neck. It is a common and severe debilitating illness that primarily affects adult females; however, it affects both sexes aged between 20 and 60 years [1]. Every type of headache contributes to the prevalence rate of CGH, which affects 2.5% of adults and ranges from 0.4% to 15% [2–4]. CGH is idiopathic in origin, but some prominent probable variables, including biopsychosocial factors, i.e., biological, social, and psychological factors, contribute to the source of CGH. Biological factors include physical health, tiresome working habits with faulty biomechanics (e.g., forward head posture), heavy lifting, degenerative disc problems, and road traffic accidents; psychological factors include anxiety, sleep disturbance, and quality of life; and social factors include family circumstances (married/divorced/widow/single) and social relationships (self-esteem/coping skill/social skill) [4–6].

The cervicogenic headache pathophysiology involves merging pain signals from multiple neck structures on top of the trigeminocervical nucleus in the brainstem [7]. This merging occurs through the top three (T1–T3) cervical nerves, which collect input from the occipital and trigeminal nerves [5]. Cervical spine malfunctioning or impairments, including disc herniation, whiplash injuries, osteoarthritis, muscle imbalances, and poor posture at the cervical level, can lead to irritation or compression of these structures, thereby activating pain pathways [1]. Moreover, activating nociceptive pathways and releasing inflammatory mediators contribute to generating and preserving pain [4].

In CGH, the frontal-temporal and orbital regions of the head are affected by the unilateral referred pain of the top three cervical spinal nerves that originates from one side of the posterior head and neck [6, 8–10]. However, a few mechanisms support the origin of neck pain and might be a reason for cervicogenic headache in forward head posture (FHP) among white-coat working professionals [11–13]. The deep cervical flexor muscle (DCFM) action line is anterior to the motion axis; it stabilizes the atlantooccipital and intervertebral joints and allows coordinated movement at the cervical spinal joint [12]. Improper functioning of these muscles may result in insufficient coordination, activation, overload, and weak support on cervical structures, progressing to neck pain and abnormal neck posture [12, 13].

Criteria for diagnosing CGH have been set by the International Headache Society (IHS) [14–16]. These requirements are as follows: (A) neck and occipital region pain are localized and may radiate to the forehead, orbital region, temples, vertex, and ears. (B) Particular neck movements or prolonged neck posture causes or exacerbates pain. (C) Presence of a minimum of 1 of the following 3 conditions: (1) limitations to resistance to passive neck movement; (2) alterations in shape, feel, tone, or reaction to active and passive stretching and contraction of the neck muscles; (3) unusual neck muscle pain. (D) At least 1 of the following is revealed by radiological examination: (1) improper flexion/extension movement; (2) an abnormal stance; (3) pathologies other than spondylosis and osteochondritis include fractures, congenital anomalies, bone cancer, and rheumatoid arthritis. Each letter heading in the IHS diagnostic criteria must be satisfied to approach a diagnosis [14, 16].

There is a pattern of typical symptoms that people with CGH experience; however, there may be some variation in their complaints. Symptoms typically start in the neck and progress to the head; furthermore, the symptoms are typically unilateral and do not switch sides. The pain can range in severity from a deep, dull ache to a heavy pressure that is either mild or severe [14, 15]. Cervicogenic headaches might start when a patient wakes up, develop over the day, or worsen, especially with prolonged neck postures or movements. Although this kind of headache can start at any age, it frequently worsens over time and may or may not be accompanied by a history of neck injury or cervical joint degenerative disease [14–16].

In previous studies, researchers have reported that people with neck pain and headaches showed a 43%–46% decrease in isometric cervical flexor muscle strength [17]. Others have demonstrated that people with cervicogenic headaches had much reduced overall cervical flexor muscle strength [18–20]. Electromyography activity for DCFM applied through a craniocervical flexion test (CCFT) confirmed the association between the weakness of DCFM and neck pain. The DCFM was recognized as a weaker performer than superficial upper cervical flexors and was suggested to be aimed at managing neck pain [7, 18, 21].

The role of manual therapy is limited in the treatment of headaches. Despite not being suitable for all forms of headaches, scientific evidence supports the use of a few manual therapies, such as cervical spinal exercises, spinal joint mobilization and manipulation, trigger point therapy, physical therapy using heat and cold packs, ultrasound, electrical stimulation, massage, acupuncture, and cognitive pain approaches, which are based on a nociceptive pain theory and aim to modulate central nervous system hypersensitivity, in the treatment of tension-type and cervicogenic headaches [22–28]. It has been demonstrated that passive cervical spine mobility or manipulation is useful in lowering or alleviating CGH. Following mobilization treatment, there are improvements in headache frequency, duration, and intensity, and a decrease in the need for headache pain medication has been recorded [25–29]. Although it has not been demonstrated that muscular tightness is a significant component of CGH, CGH has been linked to limits in cervical muscle strength, endurance, performance, and control [7, 20, 29, 30].

In CGH patients, the combination of manual treatment and muscle reeducation effectively reduced headaches and enhanced function [7, 18, 21]. In addition, a seven-week intervention study by Jull and Richardson reported that a combination of manipulative therapy and therapeutic exercises using pressure biofeedback resulted in a significant percentage (72%) of participants in the active treatment groups experiencing a decrease in headache frequency by 50% or more assessed at a 12-month follow-up. In addition, 42% of participants reported substantial relief of 80%–100%, suggesting that these findings have clinical significance [31]. Furthermore, few previous studies have reported using muscle reeducation and strengthening of the deep neck flexors to treat CGH, despite studies being conducted to evaluate the individual effectiveness of pressure biofeedback and manual treatment on headaches [7, 17–20, 27–29]. The impact of pressure biofeedback-guided DCFM strength training and manual therapy on pain has been compared in a study; [31]; however, functional impairment in people with CGH has not been compared in any study. Therefore, this study aimed to determine the efficacy of pressure biofeedback-guided DCFM strength training and manual therapy on pain and functional limitations in an individual with CGH. This study hypothesized that pressure biofeedback-guided DCFM strength training would be more effective than manual therapy in reducing pain and functional limitations in participants with CGH.

## 2. Materials and Methods

### 2.1. Study Design

This study followed a double-blinded, two-arm, parallel-group, multiple-timeline, randomized comparative design to test and compare the impact of manual therapy and pressure biofeedback-guided DCFM strength training on the outcomes of pain and functional limitations in individuals with CGH.

### 2.2. Ethical Considerations

The Ethics Committee of King Saud University approved this study (file ID: RRC-2022-04, dated: February 14, 2022), ensuring the protection of human and ethical rights in research involving human subjects. The study completed trial registration with ClinicalTrial.gov PRS (Identifier ID: NCT05692232). The 2013 revision of the Helsinki Declaration and the ethical rules of our university's Ethics Committee were followed. Each participant signed an informed consent form.

### 2.3. Sample Size

A computer program G^*∗*^ Power, version 3.10.1, was used to determine the appropriate sample size. A pilot study was conducted with 12 unmatched samples at baseline to determine the intervention effect size on the outcome score of the VAS. An a priori *t* test (unpaired *t* test) was performed, keeping the power at 0.80 (80%), the level of significance alpha set at 0.05, the mean difference at 2.25, the standard deviation difference at 0.90, the effect size at 0.5, and the correlation between the variables at 0.33. The analysis revealed that a sample of 24 individuals in each group was required to obtain an adequate sample size for this study. In addition, we accounted for a 20% dropout rate (total *N* = 60).

### 2.4. Study Setting

Sixty participants diagnosed with CGH by a consultant neurological physician were referred to the University Medical Center's outpatient physiotherapy department (OPD) for neck discomfort/pain treatment. The study was completed from February to September, 2022. Handy pamphlets, posters, and large banners were used in and around the OPD building to attract patients to participate in this study.

### 2.5. Study Participants

Sixty participants were recruited for the study based on the inclusion and exclusion criteria. The inclusion criteria were as follows: the participants exhibited unilateral head pain without side shift or bilateral head pain with a dominant side that hurts more than the other side, sustained awkward head positioning, and external pressure over the upper cervical or occipital region on the symptomatic side, recurrent CGH and chronic mechanical neck discomfort for three to twelve months, and showed positive to cervical flexion rotation test. The following conditions qualified as exclusion factors: a negative cervical flexion rotation test; subjects with a history of the following conditions: a fractured vertebral column or previous surgery on it; spinal stenosis; a prolapsed disc; TMJ dysfunction or headaches involving the autonomic nervous system; vertigo or visual disturbance; or a congenital condition of the cervical spine.

### 2.6. Procedures

**Figure 1:**
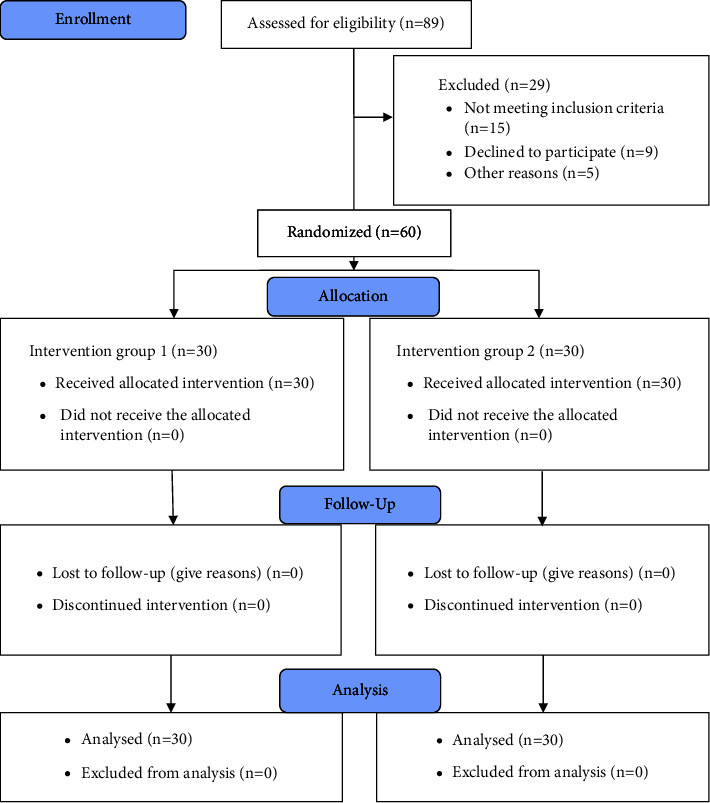
A CONSORT (2010) flow diagram shows the study procedures, such as participant enrollment, randomization, group allocation, intervention received, follow-up, and analysis.

During seven-month screenings based on this study's inclusion and exclusion criteria, sixty participants were recruited and randomly assigned to groups 1 and 2 using simple random sampling. The participants' serial numbers were assigned equally to both groups using an online, website-based randomization program (https://www.randomization.com). The randomized numbers associated with the group number were distributed to each patient in a concealed envelope to avoid selection bias by the study supervisor, who was not blinded to group allocation. Before receiving their prescribed intervention, all participants completed and submitted the consent form. An assistant physiotherapist took a baseline measurement of the demographic characteristics and results of the study at reception. Two physiotherapists were blinded to group assignment and carried out one intervention (pressure biofeedback-guided DCFM strength training) and the other (manual therapy) for each respective group. An assistant physiotherapist was also blinded to group assignment and assessed VAS and HDI at baseline (preintervention), the second week, the third week, and the fifth week after the intervention. Each outcome measurement was conducted at least twice, and the mean was incorporated into the analysis.  Figure 1 is the CONSORT (2010) flow diagram showing the study's procedures, such as participant enrollment, randomization, group allocation, follow-up, and data analysis.

### 2.7. Outcome Measures

The participants' pain intensity was measured on a 10-cm visual analog scale (VAS) with zero (0) at one end and ten (10) at the other, signifying no pain and the worst pain possible, respectively. The participants were instructed to place a mark between 0 and 10 on the VAS scale to indicate their actual amount of pain during the week/night. The VAS has been shown to be a valid and reliable instrument with the least detectable change for measuring headaches and other chronic pain [32]. The individuals' functional limitations were assessed using a beta version of the headache disability index (*β*-HDI) [33, 34]. The aim of this tool is to detect the functional and emotional challenges that participants may be experiencing due to their headaches. It is a reliable and valid 25-item self-assessment scale with two domains (functional: 12 items and emotional: 13 items) evaluating function limitations and emotional expression due to headaches. The participants were told to select “YES,” “SOMETIMES,” or “NO” for each item that relates to their subjective functional limitations and emotional feelings/expressions owing to headaches. Using this scoring system, a “YES” response to each given line was awarded 4 points, a “SOMETIMES” response was awarded 2 points, and a “NO” response was awarded 0 points. The minimum and maximum scores of the HDI range from zero (0) to one hundred (100), respectively. The sum of the scores for each item was determined by the final score; a score of score of 10 to 28 indicated a light disability, scores from 30 to 48 indicated moderate disability, scores from 50 to 68 indicated severe disability, and scores of 72 or greater indicated a complete profile [33, 34].

### 2.8. Intervention

Both groups 1 and 2 commonly received conventional treatment (i.e., moist heat pads). Group 1 performed pressure biofeedback-guided DCFM strength training, while Group 2 received manual therapy.

#### 2.8.1. Pressure Biofeedback-Guided DCFM Strength Training

Participants from Group 1 performed the DCFM strength training exercise described by Jull [11, 12]. They assumed a supine lying position, keeping the cervical spine neutral and ensuring a stabilizer pressure biofeedback unit (Chattanooga group, Hixson, TN) placed beneath the cervical lordosis. The pressure sensor was inflated at 20 mm·Hg. The therapist stood by the side and asked the participants to nod their heads slowly. As the DCFM was activated, the cervical lordosis gently flattened, and the pressure sensor measured increased pressure. The activation score is the maximum pressure that can be maintained for 10 seconds. Multiplying the target pressure by the number of successful repetitions yields the muscle's holding ability performance index. The ideal performance of the upper cervical flexor muscles would register on the pressure sensor as an increase in pressure of 10 mm·Hg held for 10 seconds, ten times on alternate days for three weeks.

#### 2.8.2. Manual Therapy

A slow, sustained elongation of muscles [35] with a holding period of 7–10 seconds and a superficial oscillatory mobilization [36] with 1-2 oscillations per second for 30 seconds per session was performed on the DCFM in a supine position and at the cervical spine (C0–C5) in a prone lying position, respectively, as part of the manual therapy. The therapist stood behind the head while performing sustained elongation of the muscles and stood by the side while performing superficial oscillatory mobilization.

#### 2.8.3. Conventional Intervention

The participants received hot water fomentation applied over the shoulder and neck region in a long sitting position for fifteen minutes per session, five days a week for four weeks [37]. The heating temperature of the hot pack was adjusted to bearable (before it could burn) by towel folding or unfolding, depending upon the participant's perception.

### 2.9. Statistical Analysis

Statistical Package for the Social Sciences (SPSS) software was used to analyze the data (IBM SPSS v.26, IBM Corp., Armonk, NY: USA). The Shapiro‒Wilk test is used to examine the distributional homogeneity of a sample. For the study of categorical data, the chi-square test was utilized. One-way analysis of variance (ANOVA): Bonferroni's multiple comparison tests were performed to assess the main effect between group factors at the different time points, within-group factors throughout the time point, and the interaction between time and group across the time point. In addition, the post hoc analysis utilized Bonferroni's multiple comparison tests to determine which group was superior to the others. The significance level alpha was set at 95%.

## 3. Result

**Figure 2:**
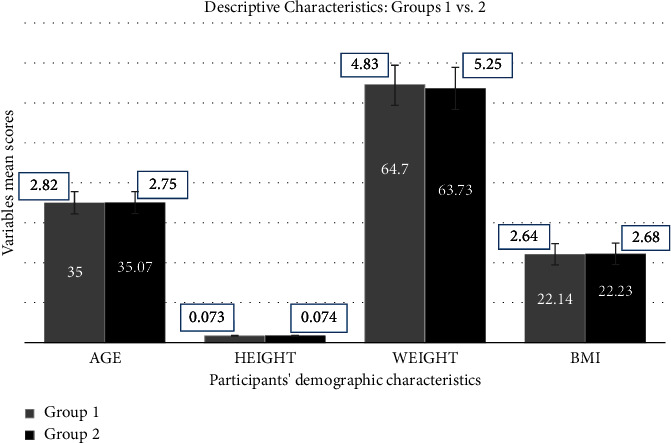
The participants' demographic characteristics mean and standard deviation scores within each group (1 vs. 2).

Sixty (females, 33; males, 27; mean age, 34.95 years) out of eighty-nine participants with chronic mechanical pain diagnosed with CGH were randomly allocated to either group (*N* = 30/group; group 1, males: 13 and females: 17; group 2, males: 14 and females: 16) in this study. A Shapiro‒Wilk test for normality reported an overall homogenous distribution of the demographic characteristics and study outcomes within each group (Table 1).  Figure 2 shows the participants' demographic characteristics mean and standard deviation scores within each group (1 vs. 2).  Table 2 provides comprehensive details of the descriptive statistics, such as sample size for each group at each interval, means, standard deviations, and 95% confidence interval for the means (lower and upper limits), minimum, maximum, first quartile, median, and third quartile.

### 3.1. Within-Group Analysis

The within-group analysis for the variables VAS and HDI revealed a statistically significant improvement (95% CI, *p* < 0.05) when comparing the postintervention values at different time intervals to the baseline scores within each group (1 and 2), as shown in Table 3.

In group 1, the VAS scores showed a significant mean difference (∆MD) when baseline was compared with the postintervention scores at different time intervals, such as VAS0-VAS1 (∆*M* = 1.433; *p*=0.001), VAS0-VAS2 (∆*M* = 3.383; *p*=0.001), VAS0-VAS3 (∆*M* = 5.367; *p*=0.001), VAS1-VAS2 (∆*M* = 1.950; *p*=0.001), VAS1-VAS3 (∆*M* = 3.933; *p*=0.001), and VAS2-VAS3 (∆*M* = 1.983; *p*=0.001). Similarly, in group 2, the VAS scores demonstrated a significant mean difference (*M*) between baseline and postintervention scores at all time intervals, including VAS0-VAS1 (*M*=.700; *p*=0.001), VAS0-VAS2 (*M* = 1.117; *p*=0.001), VAS0-VAS3 (*M* = 2.917; *p*=0.001), and VAS1–VAS3 (*M* = 2.217; *p*=0.001).

Furthermore, in group 1, the variable HDI showed a significant mean difference (∆MD) when baseline was compared with the postintervention scores at different time intervals, such as HDI0-HDI1 (∆*M* = 5.800; *p*=0.001), HDI0–HDI2 (∆*M* = 11.533; *p*=0.001), HDI0–HDI3 (∆*M* = 16.500; *p*=0.001), HDI1-HDI2 (∆*M* = 5.733; *p*=0.001), HDI1–HDI3 (∆*M* = 10.700; *p*=0.001), and HDI2-HDI3 (∆*M* = 4.967; *p*=0.001). Similarly, in group 2, the HDI scores demonstrated a significant mean difference (*M*) when comparing baseline scores to postintervention scores at all time points, including HDI0-HDI1 (*M* = 4.600; *p*=0.05), HDI0–HDI2 (*M* = 9.600; *p*=0.001), HDI0–HDI3 (*M* = 10.933; *p*=0.001), and HDI1-HDI2 (*M* = 5.000; *p*=0.05).

### 3.2. Between-Group Analysis

**Figure 3:**
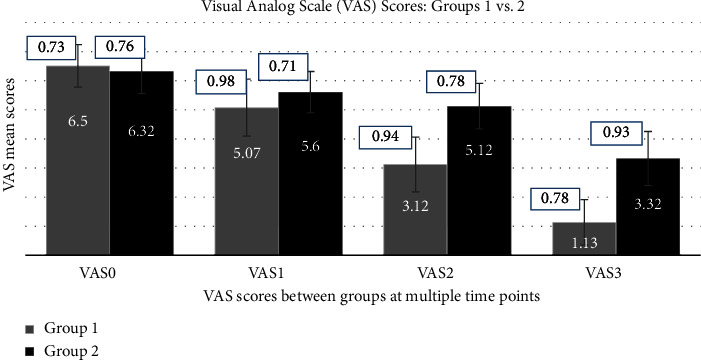
Comparison of the VAS mean scores between groups (1 vs. 2) at multiple time points.

**Figure 4:**
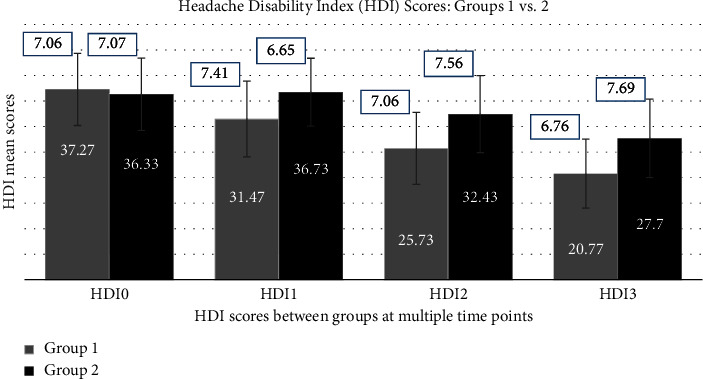
Comparison of the HDI mean scores between groups (1 vs. 2) at multiple time points.

Nevertheless, between-group (1 vs. 2) analysis of VAS and HDI outcomes revealed nonsignificant (95% CI, *p* > 0.05) mean differences at all time points, except for VAS2 and VAS3, where significant mean differences were discovered, as shown in Table 4. Moreover, Figures 3 and 4 also compare outcome mean scores between groups (1 vs. 2) at multiple time points, such as baseline and weeks 1, 2, and 3 after intervention.

Furthermore, Cohen's *d*-value indicated that the intervention effect size for reducing pain was larger in group 1 than in group 2 at weeks 2 and 3 (Table 5).

## 4. Discussion

This study compared the efficacy of pressure biofeedback-guided DCFM strength training to cervical isometric exercises on pain and functional limitation in patients with cervicogenic headaches. The results of the study indicate that DCFM strength training using pressure biofeedback was more effective at reducing pain intensity and functional limitations, thereby increasing the endurance capacity of the DCFM over a 3-week period for the treatment of CGH. A referred pain reported in any head portion generated by a primary nociceptive source in the musculoskeletal tissues innervated by cervical nerves is defined as CGH by The World Cervicogenic Headache Society (WCHS) [38].

This study provides preliminary evidence that such a trial is feasible. Manual therapy targeted to active TrPs in the sternocleidomastoid muscle may reduce headache and neck pain intensity and boost the motor function of the deep cervical flexors, PPT, and active CROM in persons with CGH and active TrPs in this muscle [39, 40]. Studies with larger sample sizes focused on long-term impacts are needed [40, 41]. The combination of physical therapy and muscle reeducation proved effective in alleviating this patient's headaches and enhancing his function [8].

Numerous researchers have investigated the anatomic basis of CGH, including the distribution of referred pain, to pinpoint the segmental location of symptomatic joints. Using fluoroscopically guided intraarticular injections, these authors demonstrated that C0-1, C1-2, and C2-3 are the segments most likely to refer pain to a location that would be experienced as a headache [8, 18, 27, 28]. This patient's articular dysfunction appeared to be localized to the upper three cervical segments, which is consistent with the findings of previous investigations on referral patterns. Manual examination of these regions replicated the patient's headache symptoms. Manual therapy has been demonstrated to reduce headache frequency, duration, and severity [21, 22, 32, 37]. Following manual therapy intervention, the researchers also discovered a decrease in analgesic use in addition to improving mobility and alleviating pain [7, 21, 22, 32, 36]. As segmental mobility increased during treatment, the provocativeness of the accessory movements diminished. By the fourth session, the altered quality of movement assessed manually was minimal, and manual assessment ceased to elicit headache symptoms.

The current scientific evidence supports the use of manual therapy in treating tension-type and cervicogenic headaches; however, the results are inconsistent. These disparate outcomes may be attributable to not all manual therapies being suited for all types of headaches, or not all headache patients will benefit from manual therapy. Based on a nociceptive pain rationale, this research provides examples of manual therapies for tension-type and cervicogenic headaches that modulate central nervous system hypersensitivity, including trigger point therapy, joint mobilization, joint manipulation, exercise, and cognitive pain methods [8, 39–41].

These results imply that DCFM strengthening exercises employing a pressure biofeedback unit are more beneficial than traditional exercise alone in lowering headache frequency in persons with CGH. The roller massage technique may be recommended to augment the initial ROM and strength of the CCF in individuals with a forward head posture [35].

### 4.1. Limitations

Despite its benefits, this study also had limitations. This study compared a manual therapy (single intervention approach) with a conventional intervention to alleviate the symptoms of patients with CGH. However, comparing a multimodal approach with a conventional intervention to manage the symptoms would have been an effective way to determine a perfect management line instead of using a single approach in patients with CGH. In addition, outcome variables, such as DCFM strength and cervical ROM, were not assessed. Therefore, future studies need to include a multimodel approach to intervention to identify the most practical and reasonable intervention for managing the symptoms, including pain, DCFM strength, ROM, and functional limitations in patients with CGH.

## 5. Conclusion

Compared with manual therapy, pressure biofeedback-guided DCFM strength training showed a greater reduction in pain intensity (VAS) at weeks two and three. However, both treatments were equally effective in lowering headache-related functional limitations in patients with CGH. While managing patients with CGH, the physical therapist should select one of the two intervention regimens based on the desired goals.

## Figures and Tables

**Table 1:** Shapiro‒Wilk test of normality for the participant distribution in both groups (*N* = 30/group).

Variables	*Group 1* (*N* = 30)				*Group 2* (*N* = 30)			
	Mean ± SD	Statistics	*df*	*p* value	Mean ± SD	Statistics	*df*	*p* value
Age (years)	35.0 ± 2.82	0.974	30	0.653	35.07 ± 2.75	0.931	30	0.052
Height (m)	1.71 ± 0.07	0.912	30	0.016^*∗*^	1.70 ± 0.07	0.972	30	0.595
Weight (kg)	64.70 ± 4.83	0.946	30	0.130	63.73 ± 5.25	0.962	30	0.344
BMI (kg/m^2^)	22.14 ± 2.59	0.871	30	0.002^*∗*^	22.23 ± 2.68	0.933	30	0.060
VAS0	6.50 ± 0.73	0.951	30	0.181	6.32 ± 0.76	0.934	30	0.062
HDI0	37.27 ± 7.06	0.959	30	0.285	36.33 ± 7.07	0.969	30	0.525

**Table 2:** Demographic characteristics of the participants from both groups (*N* = 30/group).

Variables	Mean ± SD (*N* = 30)	*95% CI for means*		Min.	Max.	Median	*Tukey's hinges*	
		Lower	Upper				1^st^ quart	3^rd^ quart
1 Age (years)	35.0 ± 2.82	33.95	36.05	29	40	35	33.00	37.00
1 Height (m)	1.71 ± 0.07	1.69	1.74	1.48	1.82	1.72	1.69	1 We
1 Weight (kg)	64.70 ± 4.83	62.90	66.50	58.00	74.00	64.50	60.00	68.00
1. BMI (kg/m^2^)	22.14 ± 2.59	21.17	23.11	18.72	29.59	22.05	20.07	23.36
2 Age (years)	35.07 ± 2.75	34.03	36.09	31	40	34	33.00	37.00
2 Height (m)	1.70 ± 0.07	1.67	1.73	1.52	1.82	1.70	1.65	2 We
2 Weight (kg)	63.73 ± 5.25	61.77	65.69	55.00	73.00	63.50	59.00	68.00
1 BMI (kg/m^2^)	22.23 ± 2.68	21.23	23.23	17.28	31.16	22.35	20.76	23.81

1: group 1; 2: group 2; SD: standard deviation; CI: confidence interval; BMI: body mass index; *N* = number of participants.

**Table 3:** Descriptive statistics of participants' outcome scores (VAS and HDI) in both groups (*N* = 30/group).

Variables	Mean ± SD (*N* = 30)	*95% CI for means*		Min.	Max.	Median	*Tukey's hinges*	
		Lower	Upper				1^st^ quart	3^rd^ quart
1 VAS0	6.50 ± 0.73	6.23	6.77	5.00	8.00	6.50	6.00	7.00
1 VAS1	5.07 ± 0.98	4.70	5.43	3.00	7.00	5.00	4.00	6.00
1 VAS2	3.12 ± 0.94	2.76	3.47	1.00	5.00	3.00	2.50	4.00
1 VAS3	1.13 ± 0.78	0.84	1.42	0.00	2.00	1.00	1.00	2.00
1 HDI0	37.27 ± 7.06	34.63	39.90	26.00	50.00	36.00	32.00	44.00
1 HDI1	31.47 ± 7.41	28.70	34.23	18.00	44.00	32.00	26.00	38.00
1 HDI2	25.73 ± 7.06	23.10	28.37	14.00	42.00	26.00	20.00	32.00
1 HDI3	20.77 ± 6.76	18.24	23.29	10.00	36.00	20.00	16.00	24.00
2 VAS0	6.32 ± 0.76	6.03	6.60	4.50	7.50	6.50	5.50	7.00
2 VAS1	5.60 ± 0.71	5.33	5.87	4.00	7.00	5.50	5.00	6.00
2 VAS2	5.12 ± 0.78	4.82	5.41	4.00	6.00	5.00	4.50	6.00
2 VAS3	3.32 ± 0.93	2.97	3.67	2.00	5.00	3.00	3.00	4.00
2 HDI0	36.33 ± 7.07	33.69	38.97	24.00	50.00	36.00	32.00	42.00
2 HDI1	36.73 ± 6.65	34.25	39.22	26.00	50.00	36.00	32.00	42.00
2 HDI2	32.43 ± 7.56	29.61	35.26	18.00	48.00	32.00	26.00	38.00
2 HDI3	27.70 ± 7.69	24.83	30.57	14.00	44.00	26.00	22.00	34.00

1: group 1; 2: group 2; SD: standard deviation; CI: confidence interval; VAS: visual analog scale; VAS0: VAS score at baseline; VAS1, 2, and 3: VAS scores at week 1^st^, 2^nd^, and 3^rd^ postintervention, respectively; HDI: headache disability index; HDI0: HDI score at baseline; HDI1, 2, and 3: HDI scores at week 1^st^, 2^nd^, and 3^rd^ postintervention, respectively; *N* = number of participants.

**Table 4:** Pairwise comparison of mean differences (treatment effect) within groups (1 and 2). Repeated measures ANOVA test: Bonferroni's multiple comparison tests, with 95% confidence interval of means.

Variables (pairwise)	*Group 1* (*n* = 30)			*Group 2* (*n* = 30)		
	Mean differences	95% CI		Mean differences	95% CI	
		*t* value	*p* value		*t* value	*p* value
VAS0-VAS1	1.433	7.709	0.001^*∗∗*^	0.700	3.981	0.001^*∗∗*^
VAS0-VAS2	3.383	18.197	0.001^*∗∗*^	1.117	6.351	0.001^*∗∗*^
VAS0-VAS3	5.367	28.864	0.001^*∗∗*^	2.917	16.589	0.001^*∗∗*^
VAS1-VAS2	1.950	10.488	0.001^*∗∗*^	0.417	2.370	0.06
VAS1-VAS3	3.933	21.155	0.001^*∗∗*^	2.217	12.607	0.001^*∗∗*^
VAS2-VAS3	1.983	10.667	0.001^*∗∗*^	1.800	10.238	0.001^*∗∗*^
HDI0-HDI1	5.800	6.660	0.001^*∗∗*^	4.600	3.042	0.05^*∗*^
HDI0-HDI2	11.533	13.244	0.001^*∗∗*^	9.600	6.349	0.001^*∗∗*^
HDI0-HDI3	16.500	18.947	0.001^*∗∗*^	10.933	7.231	0.001^*∗∗*^
HDI1-HDI2	5.733	6.583	0.001^*∗∗*^	5.000	3.307	0.01^*∗∗*^
HDI1-HDI3	10.700	12.287	0.001^*∗∗*^	6.333	4.189	0.001^*∗∗*^
HDI2-HDI3	4.967	5.703	0.001^*∗∗*^	1.333	0.882	0.19

VAS: visual analog scale; HDI: headache disability index; SD: standard deviation; ^*∗*^significant value if *p* < 0.05; ^*∗∗*^highly significant value if *p* < 0.01; CI: confidence interval; VAS1, 2, and 3: VAS scores at 1^st^, 2^nd^, and 3^rd^ week after intervention, respectively; HDI0: HDI score at baseline; HDI1, 2, and 3: HDI scores at 1^st^, 2^nd^, and 3^rd^ week after intervention, respectively; CI: confidence interval; *N* = number of participants.

**Table 5:** Between-group comparison of the variables at different timelines. One-way ANOVA test: Bonferroni's multiple comparison tests, 95% confidence interval of means.

Variables	Group 1 (*n* = 30) (Mean ± SD)	Group 2 (*n* = 30) (Mean ± SD)	*Bonferroni's multiple comparison tests*			
			∆MD	*t* values	*p* values	*d*-value
VAS0	6.50 ± 0.73	6.32 ± 0.76	0.18	0.932	1.000	n/a
VAS1	5.07 ± 0.98	5.60 ± 0.71	−0.53	2.485	1.000	0.622
VAS2	3.12 ± 0.94	5.12 ± 0.78	−2.00	9.631	0.001^*∗∗∗*^	2.343^††^
VAS3	1.13 ± 0.78	3.32 ± 0.93	−2.19	10.485	0.001^*∗∗∗*^	2.667^††^
HDI0	37.27 ± 7.06	36.33 ± 7.07	0.94	0.258	0.112	n/a
HDI1	31.47 ± 7.41	36.73 ± 6.65	−5.26	0.361	0.239	0.093
HDI2	25.73 ± 7.06	32.43 ± 7.56	−6.70	0.740	0.683	0.195
HDI3	20.77 ± 6.76	27.70 ± 7.69	−6.93	2.617	1.369	0.616

∆MD: mean differences (group 1-group 2); VAS: visual analog scale; HDI: headache disability index; SD: standard deviation; ^*∗*^significant value if *p* < 0.05; ^†^large effect size if Cohen's *d*-value > 0.8; n/a: not applicable.

## Data Availability

The dataset for the results of this study will be available from the corresponding author upon reasonable request.
